# The Reporting and Methodological Quality of Systematic Reviews Underpinning Clinical Practice Guidelines Focused on the Management of Cutaneous Melanoma: Cross-Sectional Analysis

**DOI:** 10.2196/43821

**Published:** 2023-12-07

**Authors:** Mahnoor Khalid, Bethany Sutterfield, Kirstien Minley, Ryan Ottwell, McKenna Abercrombie, Christopher Heath, Trevor Torgerson, Micah Hartwell, Matt Vassar

**Affiliations:** 1 Office of Medical Student Research Oklahoma State University Center for Health Sciences Tulsa, OK United States; 2 Oklahoma State University College of Osteopathic Medicine Tulsa, OK United States; 3 Dermatology Residency Trinity Health Ann Arbor Hospital Ypsilanti, MI United States

**Keywords:** clinical practice guidelines, clinical, cutaneous melanoma, decision making, evidence, management, melanoma, practice guideline, review, systematic review

## Abstract

**Background:**

Clinical practice guidelines (CPGs) inform evidence-based decision-making in the clinical setting; however, systematic reviews (SRs) that inform these CPGs may vary in terms of reporting and methodological quality, which affects confidence in summary effect estimates.

**Objective:**

Our objective was to appraise the methodological and reporting quality of the SRs used in CPGs for cutaneous melanoma and evaluate differences in these outcomes between Cochrane and non-Cochrane reviews.

**Methods:**

We conducted a cross-sectional analysis by searching PubMed for cutaneous melanoma guidelines published between January 1, 2015, and May 21, 2021. Next, we extracted SRs composing these guidelines and appraised their reporting and methodological rigor using the PRISMA (Preferred Reporting Items for Systematic Reviews and Meta-Analyses) and AMSTAR (A Measurement Tool to Assess Systematic Reviews) checklists. Lastly, we compared these outcomes between Cochrane and non-Cochrane SRs. All screening and data extraction occurred in a masked, duplicate fashion.

**Results:**

Of the SRs appraised, the mean completion rate was 66.5% (SD 12.29%) for the PRISMA checklist and 44.5% (SD 21.05%) for AMSTAR. The majority of SRs (19/50, 53%) were of critically low methodological quality, with no SRs being appraised as high quality. There was a statistically significant association (*P*<.001) between AMSTAR and PRISMA checklists. Cochrane SRs had higher PRISMA mean completion rates and higher methodological quality than non-Cochrane SRs.

**Conclusions:**

SRs supporting CPGs focused on the management of cutaneous melanoma vary in reporting and methodological quality, with the majority of SRs being of low quality. Increasing adherence to PRISMA and AMSTAR checklists will likely increase the quality of SRs, thereby increasing the level of evidence supporting cutaneous melanoma CPGs.

## Introduction

Clinical practice guidelines (CPGs) are high-quality, evidence-based statements that have been used by health care professionals to bridge the gap between policies, best practices, local contexts, and patient preferences [1]. Through recommendations, CPGs are beneficial to medical practices by decreasing variances and mistakes in clinical practice, reducing health care costs, and improving health outcomes [1,2]. With CPGs offering various benefits to both the clinician and the patient, it is no surprise that CPGs are heavily relied upon in clinical settings and widely supported by practicing health care professionals [3,4]. Despite their widespread use and potential benefits, concerns about the quality of CPGs exist.

Research evaluating the methodological quality and reporting clarity of systematic reviews (SRs) referenced in CPGs found variability in SR quality across various fields [5-8]. For example, CPGs focused on pediatric obesity based their recommendations primarily on low-quality SRs according to both the PRISMA (Preferred Reporting Instrument for Systematic Reviews and Meta-Analyses) and AMSTAR (A Measurement Tool to Assess Systematic Reviews) appraisal instruments [7]. PRISMA is an appraisal tool that evaluates the completeness of the reporting of SRs, while AMSTAR evaluates the methodological quality of SRs [5-8].

In dermatology, quality surveys of guidelines assessed by the Appraisal of Guidelines for Research and Evaluation Instrument (AGREE II) tool have been performed in various dermatologic conditions, including guidelines focused on the management of melanoma [9-11]. Although widely used, the AGREE II instrument was not designed to provide a comprehensive evaluation of the methodological rigor of the studies forming the guidelines and recommendations [12]. In 2020, a study found that the recommendations made by the American Academy of Dermatology (AAD) CPGs for the management of melanoma—one of the most recently published guidelines by the AAD—were supported by primarily moderate to low levels of evidence [13]. Interestingly, the lack of strong support exists despite a significant increase in published SRs and randomized controlled trials in dermatology, indicating a need for higher-quality studies [13-16]. Thus, to further improve clinical practice in dermatology, the evidence underpinning CPG recommendations needs to be rigorously developed and assessed [12,15].

With regard to limitations, the primary aim of this study was to determine the reporting and methodological quality of SRs and meta-analyses cited in CPGs for the management of cutaneous melanoma by using AMSTAR and PRISMA appraisal instruments. Our secondary aim was to evaluate the number of Cochrane SRs cited in the CPGs and explore the differences between AMSTAR and PRISMA appraisals among Cochrane SRs and non-Cochrane SRs.

## Methods

### Ethical Considerations

This study contained no human subject data and was thus exempt from institutional review board oversight.

### Transparency, Reproducibility, and Reporting

To ensure the reproducibility of this study, all data sets and analyses were publicly available on the Open Science Framework (OSF) [17]. Additionally, to further enhance reproducibility, all analyses were independently reevaluated in a masked fashion by a third-party statistician. Lastly, all search strategies, inclusion and exclusion criteria, and data extraction methods were pilot-tested a priori and adhered to this protocol.

### Outcomes

The primary objective of this study was to determine the reporting and methodological quality of SRs and meta-analyses cited in CPGs for the management of cutaneous melanoma. The methodological quality of each SR was evaluated using AMSTAR and PRISMA appraisal tools. Next, this study evaluated the number of Cochrane SRs cited in the CPG and explored the differences between AMSTAR and PRISMA appraisals among Cochrane SRs and non-Cochrane SRs.

### Identification of Clinical Practice Guidelines

To identify CPGs focused on cutaneous melanoma, a PubMed search was conducted by the author (TT). A customized search query (Multimedia Appendix 1) [1-16,18-21] was made with the aid of resources from the Canadian Agencies for Drugs and Technologies in Health [17] and the American Society of Clinical Oncology [22] and was used to identify relevant CPGs in PubMed.

After performing our search, all returned CPGs were uploaded to Rayyan QCRI (Qatar Computing Research Institute), a screening platform, to undergo inclusion criteria screening. Our definition that was used to identify CPGs was adopted from the Institute of Medicine [19]. For a CPG to be included, the following must be met: (1) the focus of the CPG was on the management of cutaneous melanoma; (2) the CPG was published between January 1, 2015, and May 21, 2021; and (3) the CPG was retrievable in English. The screening of all CPGs was performed in a masked duplicate fashion by investigators (BS and MK).

### Identification of Systematic Reviews and Meta-Analyses

Following the screening, our 2 investigators extracted all SRs and meta-analyses from each of the included CPGs in the same masked, duplicative fashion. An SR was included if the following three criteria were met: (1) the SR met the definition of an SR as defined by the PRISMA-P (Preferred Reporting Instrument for Systematic Review and Meta-Analysis Protocols) [20]; (2) the SR was available in English; and (3) it was cited in at least 1 of the included CPGs. According to PRISMA-P, “the key characteristics of an SR are (1) a clearly stated set of objectives with an explicit, reproducible methodology; (2) a systematic search that attempts to identify all studies that would meet the eligibility criteria; (3) an assessment of the validity of the findings of the included studies (eg, assessment of risk of bias and confidence in cumulative estimates); and (4) a systematic presentation and synthesis of the characteristics and findings of the included studies” [20]. Of the SRs identified during extraction, a total of 2 were not included due to not meeting the criteria for an SR as stated above.

### Training and Data Extraction

Before data extraction, investigators underwent several days of training by another investigator (TT). During this training period, investigators received training on AMSTAR and PRISMA appraisal instruments by scoring a sample of SRs according to the instrument’s instructions [21,23]. Next, both investigators discussed the results of the appraisal instruments, and additional training was provided if necessary. In addition to the AMSTAR and PRISMA appraisals, the following study characteristics were extracted from each SR: the year of publication, the population of participants, the interventions used, the number of primary studies comprising the SR, the sample size across all primary studies, and the design of each primary study. Again, all data extractions were conducted in a masked, duplicate fashion. Following data extraction, investigators were unmasked, and disagreements between data sets were resolved through group discussion. If an agreement cannot be reached, a third-party investigator (RO) is available for adjudication.

### PRISMA Checklist

PRISMA, a 27-item checklist created to increase the quality of reporting in SRs, was developed by an expert panel and scored in accordance with previous studies [5-8]. Each SR received scores based on whether full criteria were met (“yes”=1 point), whether partially met (“partial yes”=0.5 point), or whether no criteria were met (“no”=0 point) for each of the 27 items. Scores were then calculated as a proportion of the criteria met.

### AMSTAR Checklist

AMSTAR was a 16-item appraisal tool for SRs that contained either randomized or nonrandomized studies concerning health care [23], and assessment scoring was based on previous literature [5-8]. Each of the 16 items will receive a score based on the criteria met. For example, a “yes” was given if the SR met all criteria for that item, a “partial yes” if some but not all criteria were met, and a “no” if criteria were unmet. Each item was assigned a point value according to the PRISMA section. A total of 3 AMSTAR items (11, 12, and 15) are specific to SRs containing meta-analyses and are signified by an “N/A” if the SR contains no meta-analysis. Therefore, all SRs that did not include a meta-analysis were scored against 13 AMSTAR items instead of 16. Each SR receives a final critical appraisal rating of “high,” “moderate,” “low,” or “critically low” according to the AMSTAR calculator [23]. Because the AMSTAR instrument was designed for SRs that investigated a specific intervention, we were unable to appraise these SRs using the AMSTAR instrument [23].

### Secondary Analysis

A secondary analysis was performed by manually searching the Cochrane database for SRs, cross-referencing, and comparing the Cochrane SRs with SRs included in cutaneous melanoma CPGs.

### Statistical Analysis

Descriptive statistics were calculated for both PRISMA and AMSTAR completion overall and by item. We used multiple regression to determine relationships between PRISMA completion, AMSTAR appraisal, and extracted study characteristics. Lastly, to evaluate PRISMA and AMSTAR scores between Cochrane SRs and non-Cochrane SRs, a Mann-Whitney *U* test was used. Stata 16.1 (StataCorp) was used for all statistical analyses.

## Results

### General Characteristics

**Figure 1:**
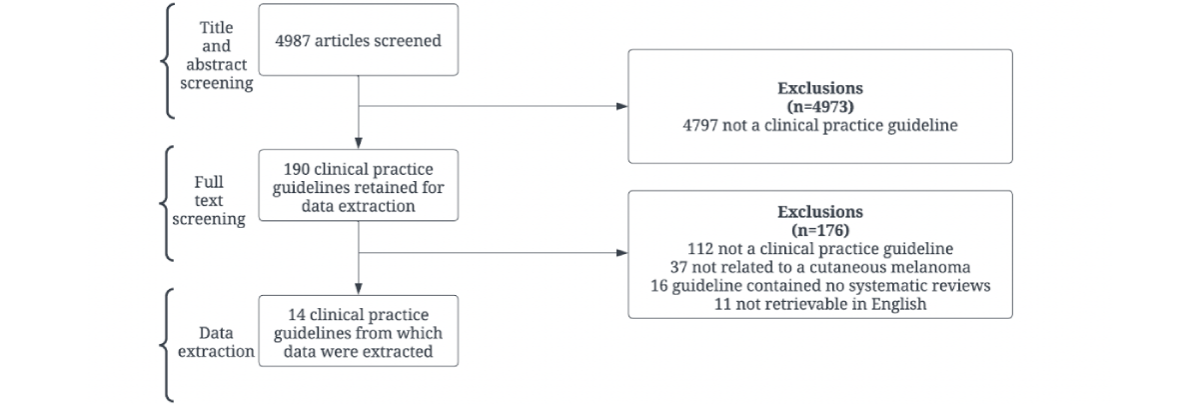
PRISMA (Preferred Reporting Items for Systematic Reviews and Meta-Analyses) flow diagram of selection process for included clinical practice guidelines (CPGs).

Our search query returned 4987 possible CPGs, of which 14 CPGs for the treatment of cutaneous melanoma were included (Figure 1). Among the 14 CPGs, 50 SRs were identified in the reference sections, and 28 of these SRs directly underpinned a guideline recommendation (Table 1). The included SRs were published between 2001 and 2018, with 70% (35/50) being published after the 2010 update of the PRISMA reporting criteria (Table 2). Of the 50 SRs, 15 (30%) were focused on diagnostic or imaging techniques, 13 (26%) covered nonsurgical interventions, 5 (10%) covered surgical interventions, and 3 (6%) were focused on both surgical and nonsurgical interventions. A total of 14 (28%) SRs did not involve an intervention. Conflict of interest statements were lacking in 10 (20%) of the 50 SRs, while 16 (32%) did not include a funding statement.

**Table 1:** Characteristics of the included clinical practice guidelines (CPGs).

CPG	Characteristics of the CPG				
	Year of publication	SRs^a^ per guideline, n	SRs supporting a guideline recommendation, n	Average PRISMA^b^ completion (%)	Average AMSTAR^c^ completion (%)
Cutaneous melanoma: ESMO^d^ Clinical Practice Guidelines for diagnosis, treatment, and follow-up [24]	2019	3	1	70	36
The updated Swiss guidelines 2016 for the treatment and follow-up of cutaneous melanoma [25]	2016	4	3	54	30
Brazilian guidelines for diagnosis, treatment, and follow-up of primary cutaneous melanoma-part II [26]	2016	6	3	56	32
Chinese Guidelines on the Diagnosis and Treatment of Melanoma (2015 Edition) [27]	2015	7	3	63	40
Diagnosis and treatment of melanoma. European consensus-based interdisciplinary guideline-Update 2016 [28]	2016	4	0	64	42
Screening for Skin Cancer: US Preventive Services Task Force Recommendation Statement [29]	2016	3	0	67	25
Updated evidence-based clinical practice guidelines for the diagnosis and management of melanoma: definitive excision margins for primary cutaneous melanoma [30]	2018	4	2	72	51
Guidelines of care for the management of primary cutaneous melanoma [31]	2018	21	14	68	44
Cutaneous Melanoma, Version 2.2019, NCCN^e^ Clinical Practice Guidelines in Oncology [32]	2019	3	0	64	28
Update on Current Treatment Recommendations for Primary Cutaneous Melanoma [33]	2019	4	3	81	62
Primary excision margins, sentinel lymph node biopsy, and completion lymph node dissection in cutaneous melanoma: a clinical practice guideline [34]	2019	1	1	77	62
Evidence-Based Clinical Practice Guidelines for the Management of Patients with Lentigo Maligna [35]	2020	3	3	70	54
SEOM^f^ clinical guideline for the management of cutaneous melanoma (2020) [36]	2021	4	2	70	59
NCCN Guidelines Insights: Melanoma: Cutaneous, Version 2.2021 [37]	2021	3	3	83	66

^a^SR: systematic review.

^b^PRISMA: Preferred Reporting Items for Systematic Reviews and Meta-Analyses.

^c^AMSTAR: A Measurement Tool to Assess Systematic Reviews.

^d^ESMO: European Society of Medical Oncology.

^e^NCCN: National Comprehensive Cancer Network.

^f^SEOM: Spanish Society of Medical Oncology.

**Table 2:** Multiple regression analysis showing the percentage of PRISMA (Preferred Reporting Items for Systematic Reviews and Meta-Analyses) completeness for systematic reviews by study characteristics.

Covariables		SRs^a^ (n=50), n (%)	Unadjusted model coefficient (SE)	*F* test (*df*)	2-tailed *t* test	*P* value	Adjusted model coefficients^b^, (SE)	Adjusted standardized coefficients	*F* test (*df*)	2-tailed *t* test	*P* value
**Year of publication^c^**				3.54 (1, 48)					F (12, 23)	—	—
	Before 2010	15 (30)	1 (reference)		—^d^	—	1 (reference)	1 (reference)		—	—
	After 2010	35 (70)	6.96 (3.7)		1.88	.07	1.18 (4.09)	0.04		0.29	.78
**Intervention type**				1.70 (4, 45)					—		
	Diagnostic or imaging technique	15 (30)	1 (reference)		—	—	1 (reference)	1 (reference)		—	—
	Nonsurgical	13 (26)	–9.37 (4.53)		–2.07	.04	–0.07 (4.42)	0.00		–0.02	.99
	No intervention^b^	14 (28)	–6.24 (4.44)		–1.40	.17	7.42 (7.54)	0.14		0.98	.34
	Surgical	5 (10)	2.54 (6.17)		0.41	.68	4.09 (5.01)	0.12		0.82	.42
	Surgical and nonsurgical	3 (6)	–9.35 (7.56)		–1.24	.22	–8.87 (6.99)	–0.20		–1.27	.22
**Conflict of interest**				3.59 (1, 48)					—		
	Statement not reported	10 (20)	1 (reference)		—	—	1 (reference)	1 (reference)		—	—
	Statement reported	40 (80)	8.03 (4.24)		1.90	.06	0.21 (4.6)	0.01		0.05	.97
**Design of included studies**				0.48 (1, 48)					—		
	Non-RCTs^e^	36 (72)	1 (reference)		—	—	1 (reference)	1 (reference)		—	—
	RCTs	14 (28)	2.69 (3.89)		0.69	.49	1.39 (3.62)	0.05		0.38	.70
**AMSTAR^f^ rating^b^ (n=36) **				25.06 (2, 33)					—		
	Critically low	19 (53)	1 (reference)		—	—	1 (reference)	1 (reference)		—	—
	Low	7 (19)	14.43 (3.56)		4.05	<.001	18.07 (4.71)	0.58		3.84	**<**.001
	Moderate	10 (28)	21.3 (3.15)		6.76	<.001	22.01 (4.56)	0.81		4.83	**<** .001
	High	0 (0)	—		—	—	—	—			—
**Funding**				0.03 (3, 46)					—		
	No funding received	12 (24)	1 (reference)		—	—	1 (reference)	1 (reference)		—	—
	No funding statement	16 (32)	–1.04 (4.84)		–0.21	.83	–3.91 (4.67)	–0.14		–0.84	.41
	Industry	2 (4)	–2.07 (9.68)		–0.21	.83	–4.67 (11.53)	–0.06		–0.41	.69
	Public or private	20 (40)	–0.14 (4.63)		–0.03	.98	–0.75 (3.57)	–0.03		–0.21	.84

^a^SR: systematic review.

^b^A total of 14 articles did not cover interventions; thus, these 14 studies were not able to be assessed by AMSTAR and were excluded from the adjusted analysis.

^c^2010 was chosen because PRISMA was first published in 2009.

^d^Not available.

^e^RCT: randomized controlled trial.

^f^AMSTAR: A Measurement Tool to Assess Systematic Reviews.

### PRISMA Completion

The mean PRISMA completion percentage of SRs was 66.5% (SD 12.3%), ranging from 37% to 89% (Multimedia Appendix 2) [38-86]. Percent completion of SRs per CPG ranged from 54% to 83% complete (Table 1). Multimedia Appendix 3 demonstrates the mean scores for all 27 items included on the PRISMA checklist. A Mann-Whitney *U* test showed that SRs published after 2010 (mean 68.5%, SD 11.7%) were not significantly better than those published before 2010 (mean 61.6%, SD 12.7%; *z*=–1.88; *P*=.06).

### AMSTAR Appraisal

Of the 50 SRs included, 14 did not cover interventions and, therefore, were unsuitable to be appraised using AMSTAR. Of the 36 remaining SRs, the mean percent completion was 44.6% (SD 21.1%), which ranged from 25% to 65.6% across CPGs (Table 1). Table S3 in Multimedia Appendix 4 demonstrates the mean scores for all items in SRs from the AMSTAR checklist. The methodological quality of these 36 SRs, according to the AMSTAR appraisal, was the following: 19 (53%) were appraised as “critically low” quality; 7 (19%) as “low” quality; 10 (28%) were “moderate” quality; and no SR received a rating of “high” quality (Multimedia Appendix 2).

### Multiple Regression

We constructed a multiple regression model to assess the relationship between PRISMA completion and the inclusion of a conflicts of interest statement, SR funding, year of publication (pre- or post-2010), intervention type, and AMSTAR rating. This model was statistically significant (*F*_12,23_=4.58; *P*<.001) and accounted for 55.1% of the variance of PRISMA completion. The model showed a statistically significant association between PRISMA completion and AMSTAR appraisals (*P*<.001), with “low” and “moderate” quality studies being more complete than “critically low” (Table 2).

### Secondary Analysis

Of the total 50 SRs, 4 (8%) were Cochrane reviews. SRs by the Cochrane group had a mean PRISMA completion of 84.7% (SD 2.1%) compared to 64.9% (SD 11.5%) among non-Cochrane studies (Multimedia Appendix 3)—a statistically significant difference (Mann-Whitney *U* test *z*=–3.10; *P*=.002). Cochrane SRs also had a higher mean AMSTAR completion (mean 86.8%, SD 4.9%) compared to non-Cochrane SRs (mean 40.0%, SD 15.1%; Multimedia Appendix 4). The Mann-Whitney *U* test also showed this difference to be statistically significant (*z*=–3.23; *P*=.001).

## Discussion

### General Findings

Our findings show that the SRs used in CPGs focused on cutaneous melanoma management vary in methodological and reporting quality, with the majority of guideline recommendations supported by poor-quality SRs. Our findings are consistent with similar studies in the fields of psychiatry, addiction medicine, cardiology, and obesity medicine [5-8]. For example, in 2017, Scott et al [6] found that the quality of SRs used in CPGs for ST-elevated myocardial infarctions was variable and reported a mean PRISMA score similar to ours. No other study in dermatology has explored the quality of SRs underpinning CPGs. However, studies have explored the methodological quality of dermatology-related SRs outside of CPGs [10,87]. In the following paragraphs, we discuss our primary findings, provide recommendations aimed at improving SRs underpinning CPGs, and review the strengths and limitations of this investigation.

The most concerning findings of this investigation were the overall poor methodological and reporting quality of SRs directly supporting a guideline recommendation, which were not shown to have improved after the publication of the revised PRISMA guidance. In fact, the vast majority of SRs that underpin a recommendation had mean PRISMA scores under 70% and were rated as having critically low methodological quality according to the AMSTAR instrument. An example of a recommendation supported by low-quality evidence can be found in the Spanish Society of Medical Oncology’s (SEOM) clinical guideline for the management of cutaneous melanoma. This guideline provides a recommendation covering positron emission tomography–computerized tomography scans based solely on a SR of low methodological quality and a mean PRISMA score of less than 70%. While the purpose of this study is not to explore the consequences of recommendations supported by poor-quality SRs, it becomes apparent how low-quality evidence supporting recommendations could impact patient care, especially in the management of diseases as dangerous as malignant melanoma.

All of the Cochrane SRs in our sample received the highest methodological quality and reporting in our sample. This finding is no surprise, as Cochrane SRs are known for their methodological rigor in producing higher-quality, less biased research results [88,89]. Interestingly, and despite the wealth of research supporting the use of Cochrane SRs, only 4 Cochrane SRs were referenced in the 14 CPGs. Additionally, only 1 of these Cochrane SRs was used to support a guideline recommendation directly. As of June 2021, there are 16 available Cochrane SRs related to the management of cutaneous melanoma, of which 4 were used in the 14 CPGs [90].

An investigation that evaluated the strength of recommendations constituting the AAD CPGs found that the majority of recommendations in this guideline were supported by weak to moderate levels of evidence [13]. Interestingly, in this same study, the authors found that the guideline with the fewest recommendations supported by strong evidence was the melanoma guideline [13]. Despite the amount of weak evidence supporting these guidelines, the problem appears to be the amount of high-quality evidence (such as SRs) in the field of dermatology. For example, a study published in 2021 found that 90% of published dermatology SRs are rated as critically low quality according to the AMSTAR instrument [87]. Similarly, Lin et al [91] found that 60% of SRs focused on atopic dermatitis received an AMSTAR methodological appraisal as either “critically low” or “low,” with only 8.8% of SRs being of “high” quality. Lastly, a study of 136 dermatology-related SRs found that the most underreported PRISMA items were protocol registration and risk of bias [10]—consistent with our findings.

### Recommendations

In an effort to improve CPGs focused on the management of cutaneous melanoma, we first recommend the use of more Cochrane SRs, as they are of the highest quality compared to non-Cochrane reviews. Next, we advocate that publishing journals update their author guidelines to require PRISMA and AMSTAR completion checklists to be submitted with manuscripts. A previous study found that mandatory PRISMA adherence was associated with improved SR reporting and methodological quality [87,92], which is similar to that found in our investigation. Furthermore, editors and peer reviewers should be provided with these checklists to ensure complete reporting and to provide revisions for improvement. Next, we advocate that journals require authors to register their protocol a priori, as registered reviews are associated with being of higher quality [93]. Finally, we advocate that evidence-based training be provided to physicians and physicians in training that focuses on these quality assessment tools. Providing this education will likely improve the knowledge and skills needed to critically appraise scientific papers included in CPGs [94].

### Strengths and Limitations

To promote the transparency and reproducibility of this study, we published our protocol on OSF a priori. Additionally, we followed the recommendations outlined in the Cochrane Handbook for Systematic Reviews of Interventions, with both investigators performing all screening and data extraction in a masked, duplicate fashion [95]. However, this study is not without limitations. Unavoidably, the PRISMA and AMSTAR checklists contain some inherent subjectivity. To mitigate subjectivity, investigators were trained before title and abstract screening on the PRISMA and AMSTAR checklists. Additionally, investigators resolved any discrepancies before final data analysis, consulting a third-party arbitrator as necessary. Another limitation of this study is only using PubMed, as it is possible some CPGs focused on the management of cutaneous melanoma could have been missed. A key limitation of this study is that the evaluation of SRs’ methodical quality does not take into account the specific needs of a CPG or whether or not the SR is relevant to the CPG. Lastly, our appraisal of SRs used the AMSTAR checklist published in 2017. Therefore, all SRs published before 2017 were only able to use the original AMSTAR checklist before publication.

### Conclusions

Our investigation found that CPGs focused on the management of cutaneous melanoma are supported by SRs that frequently underreport PRISMA items and are of critically low to low methodological quality. Additionally, we found that Cochrane SRs are of higher quality compared to non-Cochrane SRs. Future research should focus on methods to increase PRISMA and AMSTAR adherence, as doing so results in higher-quality SRs.

## Appendix

Multimedia Appendix 1 Study protocol.

Multimedia Appendix 2 Quality of systematic reviews included in clinical practice guidelines (CPGs).

Multimedia Appendix 3 PRISMA (Preferred Reporting Items for Systematic Reviews and Meta-Analyses) completeness summary for the systematic reviews comprising the 14 guidelines for the management of cutaneous melanoma.

Multimedia Appendix 4 Summary of AMSTAR-2 (A Measurement Tool to Assess Systematic Reviews) completeness scores across the 5 included guidelines.

## Abbreviations

AAD: American Academy of Dermatology
AGREE II: Appraisal of Guidelines for Research and Evaluation Instrument
AMSTAR: A Measurement Tool to Assess Systematic Reviews
CPG: clinical practice guideline
OSF: Open Science Framework
PRISMA: Preferred Reporting Items for Systematic Reviews and Meta-Analyses
PRISMA-P: Preferred Reporting Instrument for Systematic Review and Meta-Analysis Protocols
QCRI: Qatar Computing Research Institute
SEOM: Spanish Society of Medical Oncology
SR: systematic review
