# Supplementary material for: The Reporting and Methodological Quality of Systematic Reviews Underpinning Clinical Practice Guidelines Focused on the Management of Cutaneous Melanoma: Cross-Sectional Analysis
Source: JMIR Dermatol. 2023 Dec 7;6:e43821. doi: 10.2196/43821 (PMC10739238; doi:10.2196/43821)
Supplement: Multimedia Appendix 1 [file derma_v6i1e43821_app1.docx]

**Introduction:**

Clinical practice guidelines (CPGs) are high-quality, evidenced-based statements that have been used for decades by healthcare professionals to bridge the gap between policy, best practice, local contexts, and patient preferences [1]. Through evidenced-based recommendations, CPGs are essential to medical practice by offering numerous benefits such as decreasing variances in clinical practice, reducing healthcare costs, preventing mistakes and adverse events, and improving health outcomes [1,2]. With CPGs offering various benefits to both the clinician and the patient, it comes as no surprise that CPGs are heavily relied upon in clinical settings and widely supported by practicing healthcare professionals [3,4]. Despite their widespread use and potential benefits, concerns about the quality of CPGs have risen.

Research evaluating the methodological quality and reporting clarity of systematic reviews referenced in CPGs found variability in systematic review quality across various fields [5-8]. For example, CPGs focused on pediatric obesity based their recommendations primarily on systematic reviews with low methodological quality according to both the *Preferred Reporting Instrument for Systematic Reviews and Meta-Analyses* (PRISMA) and *A MeaSurement Tool to Assess systematic Reviews* (AMSTAR) appraisals instruments [7]. Similarly, Scott et al [6], found high variability in the methodological quality of systematic reviews forming the recommendations in cardiovascular CPGs.

In dermatology, quality surveys of guidelines assessed by the Appraisal of Guidelines for Research & Evaluation Instrument (AGREE II) tool have been performed in various dermatologic conditions, including guidelines focused on the management of melanoma [9-11]. Although widely used, the AGREE II instrument was not designed to provide a comprehensive and thorough evaluation of the methodological rigor of the studies forming the guidelines recommendations [12]. In 2020, a recent study found that the recommendations made in the American Academy of Dermatology (AAD) Clinical Practice Guidelines for management of melanoma —one of the most recently published guidelines by the AAD— was supported by primarily moderate to low levels of evidence [13]. Interestingly, the lack of strong support exists despite a significant increase in published systematic reviews and randomized controlled trials in dermatology, indicating a need for higher-quality studies [13-16]. Thus, to further improve clinical practice in dermatology, the evidence underpinning clinical practice guideline recommendations need to be rigorously developed and assessed [12,15].

With a need for higher quality evidence supporting melanoma guidelines and the overall lack of studies evaluating the evidence underpinning clinical practice guideline recommendations, the primary aim of this study is to determine the reporting and methodological quality of systematic reviews and meta-analyses cited in clinical practice guidelines for the management of melanoma by using AMSTAR-2 and PRISMA appraisal tools. Our secondary aim is to evaluate the number of Cochrane systematic reviews cited in the CPGs and explore the differences between AMSTAR-2 and PRISMA appraisals among Cochrane systematic reviews and non-Cochrane systematic reviews. Lastly, we will explore whether particular study characteristics are associated with higher AMSTAR-2 and PRISMA assessment scores.

**Methods:**

*Oversight, Transparency, Reproducibility, and Reporting*

This study is not using human subject data; thus is exempt from Institutional Review Board oversight. To ensure reproducibility of our study all data sets and analyses will be publicly available on Open science Framework. Additionally, to further enhance reproducibility all analyses will be independently re-evaluated in a masked fashion by a third-party statistician. Lastly, all search strategies, inclusion and exclusion criteria, and data extraction methods will be pilot tested *a priori* and adhere to this protocol.

*Outcomes:*

The primary objective of this study is to determine the reporting and methodological quality of systematic reviews and meta-analyses cited in clinical practice guidelines for the management of cutaneous melanoma. The methodological quality of each systematic review will be evaluated by using AMSTAR-2 and PRISMA appraisal tools. Next, our study will secondarily evaluate the number of Cochrane systematic reviews cited in the CPG and explore the differences between AMSTAR-2 and PRISMA appraisals among Cochrane systematic reviews and non-Cochrane systematic reviews.

*Identification of Clinical Practice Guidelines*

To identify melanoma and non-melanoma clinical practice guidelines, an initial PubMed search will be conducted by the author (TT). The following search query will be modeled from the Canadian Agencies for Drugs and Technologies in Health [17], and American Society of Clinical Oncology[(American Society of Clinical Oncology)](https://paperpile.com/c/bsGXfW/y523) which will be used to identify relevant clinical practice guidelines: ((melanoma[mh]) OR (melanoma[tiab] ) OR (melanoma[MeSH Terms])) AND (Clinical pathway[mh] OR Clinical protocol[mh] OR Consensus[mh] OR Consensus development conferences as topic[mh] OR Critical pathways[mh] OR Guidelines as topic [Mesh:NoExp] OR Practice guidelines as topic[mh] OR Health planning guidelines[mh] OR guideline[pt] OR practice guideline[pt] OR consensus development conference[pt] OR consensus development conference, NIH[pt] OR position statement*[tiab] OR policy statement*[tiab] OR practice parameter*[tiab] OR best practice*[tiab] OR standards[ti] OR guideline[ti] OR guidelines[ti] OR ((practice[tiab] OR treatment*[tiab]) AND guideline*[tiab]) OR CPG[tiab] OR CPGs[tiab] OR consensus*[tiab] OR ((critical[tiab] OR clinical[tiab] OR practice[tiab]) AND (path[tiab] OR paths[tiab] OR pathway[tiab] OR pathways[tiab] OR protocol*[tiab])) OR recommendat*[ti] OR (care[tiab] AND (standard[tiab] OR path[tiab] OR paths[tiab] OR pathway[tiab] OR pathways[tiab] OR map[tiab] OR maps[tiab] OR plan[tiab] OR plans[tiab])) OR (algorithm*[tiab] AND (screening[tiab] OR examination[tiab] OR test[tiab] OR tested[tiab] OR testing[tiab] OR assessment*[tiab] OR diagnosis[tiab] OR diagnoses[tiab] OR diagnosed[tiab] OR diagnosing[tiab])) OR (algorithm*[tiab] AND (pharmacotherap*[tiab] OR chemotherap*[tiab] OR chemotreatment*[tiab] OR therap*[tiab] OR treatment*[tiab] OR intervention*[tiab]))). After performing our initial search all returned clinical practice guidelines will be uploaded to Rayyan QCRI— a screening platform — to undergo inclusion criteria screening. Our definition that will be used to identify clinical practice guidelines will be adopted from the Institute of Medicine [18]. For a clinical practice guideline to be included the following must be met: (1) the focus of the clinical practice guideline is on the management of melanoma and non-melanoma skin cancers (2) the clinical practice guideline was published between January 1, 2015 and May, 21 2021, and (3) the clinical practice guideline is available in English. The screening of all clinical practice guidelines will be performed in a masked duplicate fashion by investigators (##, ##).

*Identification of Systematic Reviews and Meta-Analyses*

Following screening, two investigators will extract all systematic reviews and meta-analyses from each of the included clinical practice guidelines in the same masked, duplicative fashion. A systematic review will be included if the following three criteria are met: (1) The systematic review parallels the definition of a systematic review as defined by the Preferred Reporting Instrument for Systematic Reviews and Meta-Analyses Protocols (PRISMA-P) [19], (2) the systematic review is available in English, and (3) be cited in at least one of the included clinical practice guidelines.

*Training and Data Extraction :*

Prior to data extraction, investigators will undergo several days of training by investigator (TT). Through this training period investigators will receive training on AMSTAR-2 and PRISMA appraisal instruments by scoring a sample of systematic reviews according to the instrument's instructions [20,21]. Next, both investigators will discuss the results of the appraisal instruments and additional training will be provided if necessary. In addition to the AMSTAR-2 and PRISMA appraisals, the following study characteristics will be extracted from each systematic review: the year of publication, the population of participants, the interventions used, the number of primary studies comprising the systematic review, the sample size across all primary studies, and the design of each primary study. Again, all data extraction will be conducted in a masked, duplicate fashion. Following data extraction, investigators will be unmasked and disagreements between data sets will be resolved through group discussion. If an agreement can not be reached, a third party investigator (RO) is available for adjudication.

*PRISMA:*

PRISMA, a 27-item checklist created to increase the quality of reporting in systematic reviews, was developed by an expert panel and scored in accordance with previous studies [5-8]. Each systematic review will receive scores based on whether full criteria was met (‘yes’= 1 point), whether partially met (‘partial yes’ = 0.5 point) or whether no criteria were met (‘no’= 0 point) for each of the 27 items. Scores will then be calculated as a proportion of criteria met.

*AMSTAR-2:*

AMSTAR-2 is a 16-items appraisal tool for systematic reviews that contain either randomized and/or non-randomized studies concerning healthcare,[^21^](https://paperpile.com/c/bsGXfW/Pn6g5) and assessment scoring is based on previous literature [5-8]. Each of the 16 items will receive a score based on criteria met. For example, a ‘yes’ will be given if the systematic review met all criteria for that item, a ‘partial yes’ if some but not all criteria were met, and a ‘no’ if criteria was unmet. Each item is assigned a point value according to the PRISMA section. Three of the AMSTAR-2 items (11, 12, and 15) are specific to systematic reviews containing meta-analyses and are signified by an ‘N/A’ if the systematic review contains no meta-analysis. Therefore, all systematic reviews which did not include a meta-analysis will be scored against 13 AMSTAR-2 items instead of 16. The AMSTAR-2 calculator [21] Each systematic review receives a final critical appraisal rating of ‘high,’ ‘moderate,’ ‘low,’ or ‘critically low’ according to The AMSTAR-2 calculator [21].

*Secondary Analysis:*

A secondary analysis will be performed by manually searching the Cochrane database for systematic reviews, cross-referencing and comparing the Cochrane systematic reviews with systematic reviews included in melanoma and non-melanoma clinical practice guidelines.

*Statistical Analysis:*

Descriptive statistics will be calculated for both PRISMA and AMSTAR-2 assessment scores. Central tendency measures will be based upon the distributional characteristics of these assessments scores. In instances where nonnormality was observed, we will calculate and report medians and interquartile ranges. To evaluate PRISMA and AMSTAR-2 scores assessment scores, Pearson product moment correlation will be used. Next, multiple regressions will be performed to evaluate whether extracted study characteristics account for variances in PRISMA and AMSTAR-2 assessment scores. Lastly, to evaluate PRISMA and AMSTAR-2 scores between Cochrane systematic reviews and non-Cochrane systematic reviews, a Mann-Whitney U test will be used. Stata 15.1 will be used for all statistical analyses (StataCorp, LLC, College Station, TX).
