# Supplementary material for: The Reporting and Methodological Quality of Systematic Reviews Underpinning Clinical Practice Guidelines Focused on the Management of Cutaneous Melanoma: Cross-Sectional Analysis
Source: JMIR Dermatol. 2023 Dec 7;6:e43821. doi: 10.2196/43821 (PMC10739238; doi:10.2196/43821)
Supplement: Multimedia Appendix 2 [file derma_v6i1e43821_app2.docx]

**Table S1.** Quality of systematic reviews included in clinical practice guidelines (CPGs).

| **Systematic Review (PMID)*** | **PRISMA Percent Complete** | **AMSTAR 2 Percent Complete*** | **AMSTAR 2 Critical Appraisal** | **Does the SR Support a Guideline Recommendation** | **Cochrane SR** | **SR included in more than one CPG** | **CPGs the SR Supported** |
| --- | --- | --- | --- | --- | --- | --- | --- |
| Chen et al. 2001 (11735713) | 59.26 | 25.00 | Critically low | No | No | No | Screening for Skin Cancer: US Preventive Services Task Force Recommendation Statement |
| Lens et al. 2002 (12361412) | 60.00 | 37.50 | Critically low | Yes | No | No | Updated evidence-based clinical practice guidelines for the diagnosis and management of melanoma: definitive excision margins for primary cutaneous melanoma |
| Haigh et al. 2003 (14680348) | 77.78 | 50.00 | Low | Yes | No | Yes | Updated evidence-based clinical practice guidelines for the diagnosis and management of melanoma: definitive excision margins for primary cutaneous melanoma, Update on Current Treatment Recommendations for Primary Cutaneous Melanoma |
| Bafounta et al. 2004 (15522655) | 52.00 | 34.38 | Critically low | Yes | No | Yes | Brazilian guidelines for diagnosis, treatment and follow-up of primary cutaneous melanoma - Part II, Guidelines of care for the management of primary cutaneous melanoma |
| Mocellin et al. 2007 (17443001) | 66.67 | 50.00 | Low | Yes | No | No | Chinese Guidelines on the Diagnosis and Treatment of Melanoma (2015 Edition) |
| Ives et al. 2007 (18048825) | 58.00 | 37.50 | Critically low | Yes | No | No | The updated Swiss guidelines 2016 for the treatment and follow-up of cutaneous melanoma |
| Hamm et al. 2008 (18077098) | 47.83 | 23.08 | Critically low | Yes | No | No | The updated Swiss guidelines 2016 for the treatment and follow-up of cutaneous melanoma |
| Mocellin et al. 2008 (18752249) | 66.67 | 37.50 | Critically low | Yes | No | Yes | Brazilian guidelines for diagnosis, treatment and follow-up of primary cutaneous melanoma - Part II, Guidelines of care for the management of primary cutaneous melanoma |
| Krug et al. 2008 (19011184) | 76.92 | 34.62 | Low | Yes | No | No | Guidelines of care for the management of primary cutaneous melanoma |
| Rajpara et al. 2009 (19302072) | 72.00 | 65.63 | Moderate | No | No | No | Guidelines of care for the management of primary cutaneous melanoma |
| Sladden et al. 2009 (19821334) | 83.33 | 81.25 | Moderate | No | Yes | No | Primary excision margins, sentinel lymph node biopsy, and completion lymph node dissection in cutaneous melanoma: a clinical practice guideline |
| Mocellin et al. 2010 (20179267) | 72.22 | 43.75 | Critically low | Yes | No | Yes | Chinese Guidelines on the Diagnosis and Treatment of Melanoma (2015 Edition), The updated Swiss guidelines 2016 for the treatment and follow-up of cutaneous melanoma, Diagnosis and treatment of melanoma. European consensus-based interdisciplinary guideline - Update 2016, Cutaneous melanoma: ESMO Clinical Practice Guidelines for diagnosis, treatment and follow-up |
| Xing et al. 2011 (21081714) | 75.93 | 43.75 | Low | Yes | No | Yes | Chinese Guidelines on the Diagnosis and Treatment of Melanoma (2015 Edition), Update on Current Treatment Recommendations for Primary Cutaneous Melanoma, SEOM clinical guideline for the management of cutaneous melanoma (2020), Guidelines of care for the management of primary cutaneous melanoma |
| Garbe et al. 2011 (21212434) | 39.13 | 15.38 | Critically low | Yes | No | Yes | Chinese Guidelines on the Diagnosis and Treatment of Melanoma (2015 Edition), The updated Swiss guidelines 2016 for the treatment and follow-up of cutaneous melanoma, Diagnosis and treatment of melanoma. European consensus-based interdisciplinary guideline - Update 2016 |
| Valsecchi et al. 2011 (21383281) | 68.52 | 53.13 | Moderate | No | No | No | Guidelines of care for the management of primary cutaneous melanoma |
| de Rosa et al. 2011 (21540313) | 64.00 | 34.62 | Critically low | Yes | No | No | Primary excision margins, sentinel lymph node biopsy, and completion lymph node dissection in cutaneous melanoma: a clinical practice guideline |
| Petrella et al. 2012 (22245520) | 50.00 | 37.50 | Critically low | No | No | No | Chinese Guidelines on the Diagnosis and Treatment of Melanoma (2015 Edition) |
| Cromwell et al. 2012 (22914178) | 54.35 | 23.08 | Critically low | No | No | No | Guidelines of care for the management of primary cutaneous melanoma |
| Vourc'h-Jourdain et al. 2013 (23182059) | 63.04 | 23.08 | Low | No | No | No | Brazilian guidelines for diagnosis, treatment and follow-up of primary cutaneous melanoma - Part II |
| Schröer-Günther et al. 2012 (23237499) | 69.23 | 50.00 | Low | Yes | No | Yes | Chinese Guidelines on the Diagnosis and Treatment of Melanoma (2015 Edition), SEOM clinical guideline for the management of cutaneous melanoma (2020) |
| Minkis et al. 2013 (23357570) | 64.00 | 31.25 | Critically low | Yes | No | No | Guidelines of care for the management of primary cutaneous melanoma |
| Stevenson et al. 2013 (24282659) | 66.00 | 59.38 | Moderate | No | No | No | Guidelines of care for the management of primary cutaneous melanoma |
| Flaherty et al. 2014 (24485879) | 68.52 | 28.13 | Critically low | No | No | No | Cutaneous melanoma: ESMO Clinical Practice Guidelines for diagnosis, treatment and follow-up |
| Matsuda et al. 2015 (25119122) | 66.67 | 37.50 | Critically low | Yes | No | No | Chinese Guidelines on the Diagnosis and Treatment of Melanoma (2015 Edition) |
| Flaherty et al. 2014 (24485879) | 82.61 | 92.31 | Moderate | No | Yes | Yes | Evidence-Based Clinical Practice Guidelines for the Management of Patients with Lentigo Maligna, Guidelines of care for the management of primary cutaneous melanoma |
| Kyrgidis et al. 2015 (25978975) | 86.96 | 89.29 | Moderate | No | Yes | No | Update on Current Treatment Recommendations for Primary Cutaneous Melanoma |
| Mora et al. 2015 (26088690) | 58.70 | 30.77 | Critically low | Yes | No | Yes | Evidence-Based Clinical Practice Guidelines for the Management of Patients with Lentigo Maligna, Guidelines of care for the management of primary cutaneous melanoma |
| Read et al. 2016 (26299846) | 47.83 | 30.77 | Critically low | Yes | No | Yes | Evidence-Based Clinical Practice Guidelines for the Management of Patients with Lentigo Maligna, Guidelines of care for the management of primary cutaneous melanoma |
| Bertrand et al. 2015 (26337719) | 79.63 | 59.38 | Low | No | No | No | Cutaneous Melanoma, Version 2.2019, NCCN Clinical Practice Guidelines in Oncology |
| Wheatley et al. 2016 (26563920) | 79.63 | 65.63 | Moderate | Yes | No | Yes | Diagnosis and treatment of melanoma. European consensus-based interdisciplinary guideline - Update 2016, Primary excision margins, sentinel lymph node biopsy, and completion lymph node dissection in cutaneous melanoma: a clinical practice guideline, Updated evidence-based clinical practice guidelines for the diagnosis and management of melanoma: definitive excision margins for primary cutaneous melanoma, |
| Cordeiro et al. 2016 (26932710) | 82.00 | 65.63 | Moderate | Yes | No | Yes | Guidelines of care for the management of primary cutaneous melanoma, Update on Current Treatment Recommendations for Primary Cutaneous Melanoma, Primary excision margins, sentinel lymph node biopsy, and completion lymph node dissection in cutaneous melanoma: a clinical practice guideline, NCCN Guidelines® Insights: Melanoma: Cutaneous, Version 2.2021 |
| Abdel-Wahab et al. 2016 (27472273) | 67.39 | 42.31 | Critically low | Yes | No | No | Guidelines of care for the management of primary cutaneous melanoma |
| Tio et al. 2017 (27987308) | 89.13 | 61.54 | Moderate | Yes | No | Yes | Evidence-Based Clinical Practice Guidelines for the Management of Patients with Lentigo Maligna, Guidelines of care for the management of primary cutaneous melanoma |
| Ives et al. 2017 (28692949) | 57.41 | 15.63 | Critically low | No | No | No | Cutaneous Melanoma, Version 2.2019, NCCN Clinical Practice Guidelines in Oncology |
| Wang et al. 2018 (30242316) | 54.00 | 9.38 | Critically low | No | No | No | Cutaneous Melanoma, Version 2.2019, NCCN Clinical Practice Guidelines in Oncology |
| Dinnes et al. 2018 (30521681) | 86.00 | 84.38 | Moderate | Yes | Yes | No | SEOM clinical guideline for the management of cutaneous melanoma (2020) |
| Watt et al. 2004 (15253185)* | 47.83 | - | - | No | No | No | Brazilian guidelines for diagnosis, treatment and follow-up of primary cutaneous melanoma - Part II |
| Gandini et al. 2005 (15617989)* | 62.96 | - | - | No | No | No | SEOM clinical guideline for the management of cutaneous melanoma (2020) |
| Gandini et al. 2005 (16125929)* | 55.56 | - | - | No | No | No | SEOM clinical guideline for the management of cutaneous melanoma (2020) |
| Krengel et al. 2006 (16792745)* | 36.96 | - | - | Yes | No | No | Brazilian guidelines for diagnosis, treatment and follow-up of primary cutaneous melanoma - Part II |
| Gandini et al. 2011 (21620689)* | 66.67 | - | - | No | No | No | Guidelines of care for the management of primary cutaneous melanoma |
| Ensslin et al. 2013 (23981682)* | 64.00 | - | - | Yes | No | No | Guidelines of care for the management of primary cutaneous melanoma |
| Vuong et al. 2014 (24522401)* | 67.39 | - | - | No | No | No | Brazilian guidelines for diagnosis, treatment and follow-up of primary cutaneous melanoma - Part II |
| Caini et al. 2014 (24680127)* | 82.00 | - | - | No | No | No | Update on Current Treatment Recommendations for Primary Cutaneous Melanoma |
| Teulings et al. 2015 (25605840)* | 66.67 | - | - | Yes | No | No | Guidelines of care for the management of primary cutaneous melanoma |
| Byrom et al. 2015 (25690106)* | 68.00 | - | - | Yes | No | No | Guidelines of care for the management of primary cutaneous melanoma |
| Byrom et al. 2015 (26177116)* | 62.00 | - | - | Yes | No | No | Guidelines of care for the management of primary cutaneous melanoma |
| Ribero et al. 2015 (26332402)* | 83.33 | - | - | Yes | No | Yes | NCCN Guidelines® Insights: Melanoma: Cutaneous, Version 2.2021, Guidelines of care for the management of primary cutaneous melanoma |
| Wernli et al. 2016 (27458949)* | 74.00 | - | - | No | No | No | Screening for Skin Cancer: US Preventive Services Task Force Recommendation Statement |
| Gualano et al. 2017 (28386936)* | 72.00 | - | - | No | No | No | Guidelines of care for the management of primary cutaneous melanoma |
| **Overall Percent Completed; Mean (SD)** | **66.45, (12.29)** | **32.08, (26.93)** |  |  |  |  |  |
| aPRISMA and AMSTAR-2 scores are percent of criteria met 14 articles did not cover interventions; thus, these 14 studies were not able to be assessed by AMSTAR-2 and were excluded from adjusted analysis. | | | | | | | |
