# Supplementary material for: The Reporting and Methodological Quality of Systematic Reviews Underpinning Clinical Practice Guidelines Focused on the Management of Cutaneous Melanoma: Cross-Sectional Analysis
Source: JMIR Dermatol. 2023 Dec 7;6:e43821. doi: 10.2196/43821 (PMC10739238; doi:10.2196/43821)
Supplement: Multimedia Appendix 3 [file derma_v6i1e43821_app3.docx]

**Table S2.** PRISMA (Preferred Reporting Items for Systematic Reviews and Meta-Analyses) completeness summary for the systematic reviews comprising the 14 guidelines for the management of cutaneous melanoma.

| **PRISMA Item** | **Cutaneous melanoma: ESMO CPGs for diagnosis, treatment and follow-up**  **(SRs=2)** | **The updated Swiss guidelines 2016 for the treatment and follow-up of cutaneous melanoma**  **(SRs=4)** | **Brazilian guidelines for diagnosis, treatment and follow-up of primary cutaneous melanoma-Part II**  **(SRs=6)** | **Chinese Guidelines on the Diagnosis and Treatment of Melanoma (2015 Edition)**  **(SRs=7)** |
| --- | --- | --- | --- | --- |
|  | Item Score  Mean, (SD) | Item Score  Mean, (SD) | Item Score  Mean, (SD) | Item Score  Mean, (SD) |
| 1. Title: systematic review, meta-analysis, both? | 0.75, (0.35) | 0.88, (0.25) | 0.83, (0.26) | 0.71, (0.27) |
| 2. Abstract: structured summary? | 0.5, (0) | 0.5, (0) | 0.58, (0.2) | 0.57, (0.19) |
| 3. Introduction: rationale for review? | 1, (0) | 1, (0) | 1, (0) | 1, (0) |
| 4. Introduction: explicit statement of objectives? | 1, (0) | 0.5, (0.58) | 0.58, (0.38) | 0.79, (0.39) |
| 5. Methods: protocol and registration? | 0, (0) | 0.13, (0.25) | 0.08, (0.2) | 0.07, (0.19) |
| 6. Methods: eligibility criteria? | 1, (0) | 0.5, (0.41) | 0.92, (0.2) | 0.79, (0.39) |
| 7. Methods: information sources? | 0.75, (0.35) | 0.88, (0.25) | 1, (0) | 0.86, (0.24) |
| 8. Methods: full search strategy? | 0.75, (0.35) | 0.5, (0) | 0.5, (0.32) | 0.43, (0.19) |
| 9. Methods: study selection process? | 0.5, (0.71) | 0.25, (0.5) | 0.5, (0.55) | 0.43, (0.53) |
| 10. Methods: data collection process? | 0.5, (0.71) | 0.5, (0.58) | 0.67, (0.52) | 0.29, (0.49) |
| 11. Methods: data items to be extracted? | 1, (0) | 0.5, (0.58) | 0.58, (0.49) | 0.57, (0.53) |
| 12. Methods: risk of bias of individual studies? | 0, (0) | 0, (0) | 0.17, (0.41) | 0.43, (0.53) |
| 13. Methods: summary measures? | 0.75, (0.35) | 0.63, (0.48) | 0.25, (0.42) | 0.5, (0.41) |
| 14. Methods: synthesis of results? | 0.5, (0.71) | 1, (0) | 0.5, (0.71) | 0.67, (0.52) |
| 15. Methods: risk of bias across studies? | 0.5, (0.71) | 0.25, (0.5) | 0.17, (0.41) | 0.57, (0.53) |
| 16. Methods: additional analyses? | 0.75, (0.35) | 0.5, (0) | 1, (0) | 0.63, (0.48) |
| 17. Results: study selection? | 1, (0) | 0.5, (0.58) | 0.58, (0.49) | 0.5, (0.5) |
| 18. Results: study characteristics? | 1, (0) | 0.88, (0.25) | 1, (0) | 0.93, (0.19) |
| 19. Results: risk of bias within studies? | 0, (0) | 0, (0) | 0, (0) | 0.29, (0.49) |
| 20. Results: results of individual studies? | 1, (0) | 1, (0) | 0.58, (0.2) | 1, (0) |
| 21. Results: synthesis of results? | 1, (0) | 1, (0) | 0.75, (0.35) | 1, (0) |
| 22. Results: risk of bias across studies? | 0.5, (0.71) | 0.25, (0.5) | 0.17, (0.41) | 0.57, (0.53) |
| 23. Results: results of additional analyses? | 1, (0) | 1, (0) | 1, (0) | 1, (0) |
| 24. Discussion: summary of evidence? | 0.5, (0) | 0.63, (0.25) | 0.83, (0.26) | 0.71, (0.27) |
| 25. Discussion: study limitations? | 0.75, (0.35) | 0.13, (0.25) | 0.5, (0.45) | 0.64, (0.48) |
| 26. Discussion: conclusions? | 1, (0) | 0.75, (0.29) | 0.83, (0.26) | 0.86, (0.24) |
| 27. Funding: funding sources and role of funders? | 1, (0) | 1, (0) | 0, (0) | 0.5, (0.5) |
| Overall PRISMA completeness: | 70.37, (2.62) | 54.29, (14.22) | 55.65, (12.15) | 62.83, (13.27) |

| **Supplementary Table 2. Continued.** | | | | |
| --- | --- | --- | --- | --- |
| **PRISMA Item** | **Diagnosis and treatment of melanoma. European consensus-based interdisciplinary guideline - Update 2016**  **(SRs=3)** | **Screening for Skin Cancer: US Preventive Services Task Force Recommendation Statement**  **(SRs=2)** | **Updated evidence-based CPGs for the diagnosis and management of melanoma: definitive excision margins for primary cutaneous melanoma**  **(SRs=3)** | **Guidelines of care for the management of primary cutaneous melanoma**  **(SRs=20)** |
| 1. Title: systematic review, meta-analysis, both? | 0.83, (0.29) | 0.75, (0.35) | 0.67, (0.29) | 0.88, (0.22) |
| 2. Abstract: structured summary? | 0.5, (0) | 0.75, (0.35) | 0.67, (0.29) | 0.5, (0) |
| 3. Introduction: rationale for review? | 1, (0) | 1, (0) | 1, (0) | 1, (0) |
| 4. Introduction: explicit statement of objectives? | 0.5, (0.5) | 1, (0) | 0.83, (0.29) | 0.73, (0.3) |
| 5. Methods: protocol and registration? | 0.33, (0.58) | 0, (0) | 0.67, (0.58) | 0.2, (0.25) |
| 6. Methods: eligibility criteria? | 0.83, (0.29) | 1, (0) | 1, (0) | 0.88, (0.32) |
| 7. Methods: information sources? | 0.83, (0.29) | 1, (0) | 1, (0) | 0.88, (0.22) |
| 8. Methods: full search strategy? | 0.5, (0) | 0.75, (0.35) | 0.67, (0.29) | 0.55, (0.22) |
| 9. Methods: study selection process? | 0, (0) | 1, (0) | 0.33, (0.58) | 0.55, (0.48) |
| 10. Methods: data collection process? | 0.67, (0.58) | 1, (0) | 1, (0) | 0.58, (0.49) |
| 11. Methods: data items to be extracted? | 0.67, (0.58) | 0.5, (0.71) | 0.83, (0.29) | 0.93, (0.18) |
| 12. Methods: risk of bias of individual studies? | 0, (0) | 0.5, (0.71) | 0, (0) | 0.5, (0.49) |
| 13. Methods: summary measures? | 0.33, (0.29) | 0, (0) | 0.83, (0.29) | 0.55, (0.48) |
| 14. Methods: synthesis of results? | 1, (0) | 1, (0) | 0.67, (0.58) | 0.67, (0.49) |
| 15. Methods: risk of bias across studies? | 0.67, (0.58) | 0, (0) | 0.67, (0.58) | 0.3, (0.47) |
| 16. Methods: additional analyses? | 0.75, (0.35) | 0.5, (0.71) | 1, (0) | 0.8, (0.45) |
| 17. Results: study selection? | 0.67, (0.58) | 0.75, (0.35) | 0.5, (0.5) | 0.7, (0.41) |
| 18. Results: study characteristics? | 1, (0) | 1, (0) | 1, (0) | 0.95, (0.15) |
| 19. Results: risk of bias within studies? | 0.33, (0.58) | 0.5, (0.71) | 0.67, (0.58) | 0.43, (0.49) |
| 20. Results: results of individual studies? | 1, (0) | 0.25, (0.35) | 1, (0) | 0.85, (0.24) |
| 21. Results: synthesis of results? | 1, (0) | 0, (0) | 1, (0) | 0.97, (0.13) |
| 22. Results: risk of bias across studies? | 0.67, (0.58) | 0, (0) | 0.67, (0.58) | 0.35, (0.46) |
| 23. Results: results of additional analyses? | 1, (0) | 1, (0) | 1, (0) | 0.67, (0.52) |
| 24. Discussion: summary of evidence? | 0.67, (0.29) | 0.5, (0.71) | 0.83, (0.29) | 0.75, (0.26) |
| 25. Discussion: study limitations? | 0.17, (0.29) | 1, (0) | 0.17, (0.29) | 0.85, (0.29) |
| 26. Discussion: conclusions? | 1, (0) | 1, (0) | 0.83, (0.29) | 0.85, (0.24) |
| 27. Funding: funding sources and role of funders? | 1, (0) | 1, (0) | 0.33, (0.58) | 0.7, (0.44) |
| Overall PRISMA completeness: | 63.66, (21.56) | 66.63, (10.42) | 72.47, (10.84) | 67.87, (10.42) |

| **Supplementary Table 2.** Continued | | | | |
| --- | --- | --- | --- | --- |
| **PRISMA Item** | **Cutaneous Melanoma, Version 2.2019, NCCN CPGs in Oncology**  **(SRs=3)** | **Update on Current Treatment Recommendations for Primary Cutaneous Melanoma**  **(SRs=5)** | **Primary excision margins, sentinel lymph node biopsy, and completion lymph node dissection in cutaneous melanoma: a CPG**  **(SRs=4)** | **Evidence-Based CPGs for the Management of Patients with Lentigo Maligna**  **(SRs=4)** |
| 1. Title: systematic review, meta-analysis, both? | 0.83, (0.29) | 0.7, (0.45) | 0.63, (0.48) | 0.75, (0.5) |
| 2. Abstract: structured summary? | 0.5, (0) | 0.5, (0) | 0.63, (0.25) | 0.5, (0) |
| 3. Introduction: rationale for review? | 1, (0) | 1, (0) | 1, (0) | 1, (0) |
| 4. Introduction: explicit statement of objectives? | 0.5, (0) | 1, (0) | 0.63, (0.25) | 0.88, (0.25) |
| 5. Methods: protocol and registration? | 0, (0) | 0.6, (0.42) | 0.63, (0.48) | 0.38, (0.48) |
| 6. Methods: eligibility criteria? | 0.83, (0.29) | 1, (0) | 1, (0) | 1, (0) |
| 7. Methods: information sources? | 0.83, (0.29) | 1, (0) | 0.88, (0.25) | 0.88, (0.25) |
| 8. Methods: full search strategy? | 0.5, (0.5) | 0.8, (0.27) | 0.75, (0.29) | 0.63, (0.25) |
| 9. Methods: study selection process? | 0.33, (0.58) | 0.6, (0.55) | 0.25, (0.5) | 0.75, (0.5) |
| 10. Methods: data collection process? | 0.5, (0.5) | 0.8, (0.45) | 1, (0) | 0.5, (0.58) |
| 11. Methods: data items to be extracted? | 0.67, (0.58) | 1, (0) | 1, (0) | 1, (0) |
| 12. Methods: risk of bias of individual studies? | 0.33, (0.58) | 0.8, (0.45) | 0.75, (0.5) | 0.5, (0.58) |
| 13. Methods: summary measures? | 0.17, (0.29) | 0.9, (0.22) | 0.5, (0.41) | 0.5, (0.58) |
| 14. Methods: synthesis of results? | 0.5, (0.5) | 0.5, (0.58) | 1, (0) | 0, (0) |
| 15. Methods: risk of bias across studies? | 0.33, (0.58) | 0.8, (0.45) | 0.25, (0.5) | 0.38, (0.48) |
| 16. Methods: additional analyses? | 0.5, (0.71) | 1, (0) | 1, (0) | 0, (0) |
| 17. Results: study selection? | 1, (0) | 0.6, (0.55) | 0.88, (0.25) | 0.5, (0.41) |
| 18. Results: study characteristics? | 0.83, (0.29) | 1, (0) | 0.88, (0.25) | 1, (0) |
| 19. Results: risk of bias within studies? | 0.33, (0.58) | 0.8, (0.45) | 0.75, (0.5) | 0.5, (0.58) |
| 20. Results: results of individual studies? | 1, (0) | 0.9, (0.22) | 1, (0) | 0.63, (0.25) |
| 21. Results: synthesis of results? | 1, (0) | 1, (0) | 1, (0) | 0, (0) |
| 22. Results: risk of bias across studies? | 0.33, (0.58) | 0.6, (0.55) | 0.25, (0.5) | 0.25, (0.5) |
| 23. Results: results of additional analyses? | 1, (0) | 1, (0) | 1, (0) | 0, (0) |
| 24. Discussion: summary of evidence? | 0.83, (0.29) | 0.8, (0.27) | 0.75, (0.29) | 0.75, (0.29) |
| 25. Discussion: study limitations? | 1, (0) | 0.8, (0.27) | 0.75, (0.5) | 0.88, (0.25) |
| 26. Discussion: conclusions? | 1, (0) | 0.9, (0.22) | 1, (0) | 0.88, (0.25) |
| 27. Funding: funding sources and role of funders? | 0.67, (0.58) | 0.6, (0.55) | 1, (0) | 1, (0) |
| Overall PRISMA completeness: | 63.68, (13.92) | 80.93, (4.29) | 77.24, (8.96) | 69.57, (19.52) |

| **Supplementary Table 2.** Continued. | | | | | | |
| --- | --- | --- | --- | --- | --- | --- |
| **PRISMA Item** | **Evidence-Based Clinical Practice Guidelines for the Management of Patients with Lentigo Maligna**  **(SRs=4)** | **SEOM clinical guideline for the management of cutaneous melanoma (2020)**  **(SRs=5)** | **NCCN Guidelines® Insights: Melanoma: Cutaneous, Version 2.2021**  **(SRs=2)** | **Overall**  **(SRs=50)** | **Cochrane**  **(SRs=4)** | **Non-Cochrane**  **(SRs=46)** |
| 1. Title: systematic review, meta-analysis, both? | 0.75, (0.5) | 0.4, (0.22) | 1, (0) | 0.75, (0.32) | 0, (0) | 0.82, (0.24) |
| 2. Abstract: structured summary? | 0.5, (0) | 0.5, (0) | 0.5, (0) | 0.55, (0.15) | 0.63, (0.25) | 0.54, (0.14) |
| 3. Introduction: rationale for review? | 1, (0) | 1, (0) | 1, (0) | 1, (0) | 1, (0) | 1, (0) |
| 4. Introduction: explicit statement of objectives? | 0.88, (0.25) | 0.7, (0.27) | 1, (0) | 0.72, (0.31) | 0.75, (0.29) | 0.72, (0.31) |
| 5. Methods: protocol and registration? | 0.38, (0.48) | 0.2, (0.45) | 0.5, (0) | 0.23, (0.35) | 1, (0) | 0.16, (0.28) |
| 6. Methods: eligibility criteria? | 1, (0) | 0.9, (0.22) | 1, (0) | 0.86, (0.3) | 1, (0) | 0.85, (0.31) |
| 7. Methods: information sources? | 0.88, (0.25) | 0.9, (0.22) | 1, (0) | 0.9, (0.2) | 1, (0) | 0.89, (0.21) |
| 8. Methods: full search strategy? | 0.63, (0.25) | 0.5, (0.35) | 0.75, (0.35) | 0.57, (0.27) | 0.88, (0.25) | 0.54, (0.25) |
| 9. Methods: study selection process? | 0.75, (0.5) | 0.4, (0.55) | 0.5, (0.71) | 0.54, (0.49) | 1, (0) | 0.5, (0.49) |
| 10. Methods: data collection process? | 0.5, (0.58) | 0.4, (0.55) | 0.75, (0.35) | 0.6, (0.48) | 1, (0) | 0.57, (0.49) |
| 11. Methods: data items to be extracted? | 1, (0) | 0.8, (0.45) | 1, (0) | 0.77, (0.39) | 1, (0) | 0.75, (0.4) |
| 12. Methods: risk of bias of individual studies? | 0.5, (0.58) | 0.8, (0.27) | 1, (0) | 0.44, (0.48) | 1, (0) | 0.39, (0.47) |
| 13. Methods: summary measures? | 0.5, (0.58) | 0.8, (0.45) | 1, (0) | 0.52, (0.45) | 0.75, (0.29) | 0.5, (0.46) |
| 14. Methods: synthesis of results? | 0, (0) | 0.6, (0.55) | 1, (0) | 0.73, (0.44) | 1, (0) | 0.71, (0.45) |
| 15. Methods: risk of bias across studies? | 0.38, (0.48) | 0.6, (0.55) | 0.5, (0.71) | 0.35, (0.48) | 0.38, (0.48) | 0.35, (0.48) |
| 16. Methods: additional analyses? | 0, (0) | 1, (0) | 1, (0) | 0.78, (0.41) | 1, (0) | 0.76, (0.42) |
| 17. Results: study selection? | 0.5, (0.41) | 0.5, (0.5) | 1, (0) | 0.7, (0.4) | 0.88, (0.25) | 0.68, (0.41) |
| 18. Results: study characteristics? | 1, (0) | 0.9, (0.22) | 1, (0) | 0.94, (0.16) | 1, (0) | 0.93, (0.17) |
| 19. Results: risk of bias within studies? | 0.5, (0.58) | 0.6, (0.55) | 1, (0) | 0.35, (0.48) | 1, (0) | 0.29, (0.45) |
| 20. Results: results of individual studies? | 0.63, (0.25) | 1, (0) | 1, (0) | 0.86, (0.25) | 1, (0) | 0.85, (0.26) |
| 21. Results: synthesis of results? | 0, (0) | 1, (0) | 1, (0) | 0.96, (0.19) | 1, (0) | 0.95, (0.19) |
| 22. Results: risk of bias across studies? | 0.25, (0.5) | 0.8, (0.45) | 0.5, (0.71) | 0.34, (0.47) | 0, (0) | 0.37, (0.48) |
| 23. Results: results of additional analyses? | 0, (0) | 1, (0) | 0, (0) | 0.91, (0.29) | 1, (0) | 0.9, (0.3) |
| 24. Discussion: summary of evidence? | 0.75, (0.29) | 0.7, (0.27) | 0.75, (0.35) | 0.75, (0.27) | 1, (0) | 0.73, (0.27) |
| 25. Discussion: study limitations? | 0.88, (0.25) | 0.6, (0.55) | 1, (0) | 0.69, (0.42) | 1, (0) | 0.66, (0.42) |
| 26. Discussion: conclusions? | 0.88, (0.25) | 0.8, (0.27) | 0.75, (0.35) | 0.86, (0.23) | 1, (0) | 0.85, (0.23) |
| 27. Funding: funding sources and role of funders? | 1, (0) | 0.6, (0.55) | 0.5, (0.71) | 0.65, (0.47) | 1, (0) | 0.62, (0.47) |
| Overall PRISMA completeness: | 69.57, (19.52) | 69.94, (11.72) | 82.67, (0.94) | 66.45, (12.29) | 84.72, (2.08) | 64.87, (11.49) |
