# Supplementary material for: The Reporting and Methodological Quality of Systematic Reviews Underpinning Clinical Practice Guidelines Focused on the Management of Cutaneous Melanoma: Cross-Sectional Analysis
Source: JMIR Dermatol. 2023 Dec 7;6:e43821. doi: 10.2196/43821 (PMC10739238; doi:10.2196/43821)
Supplement: Multimedia Appendix 4 [file derma_v6i1e43821_app4.docx]

| Supplementary Table 3. Summary of AMSTAR-2 Completeness Scores Across the Five Included Guidelines | | | | |
| --- | --- | --- | --- | --- |
| **AMSTAR-2 Item** | **Cutaneous melanoma: ESMO CPGs for diagnosis, treatment and follow-up**  **(SRs=2)** | **The updated Swiss guidelines 2016 for the treatment and follow-up of cutaneous melanoma**  **(SRs=4)** | **Brazilian guidelines for diagnosis, treatment and follow-up of primary cutaneous melanoma - Part II**  **(SRs=3)** | **Chinese Guidelines on the Diagnosis and Treatment of Melanoma (2015 Edition)**  **(SRs=7)** |
|  | Item Score,  Mean (SD) | Item Score,  Mean (SD) | Item Score,  Mean (SD) | Item Score,  Mean (SD) |
| 1) Did the research questions and inclusion criteria for the review include the components of PICO? | 0.5, (0.71) | 0.75, (0.5) | 0.67, (0.58) | 1, (0) |
| 2) Did the SR contain an explicit statement that the methods were established prior to the SR and did the report justify any significant deviations from the protocol? | 0, (0) | 0, (0) | 0, (0) | 0, (0) |
| 3) Did the SR authors explain their selection of the study designs for inclusion? | 0, (0) | 0.25, (0.5) | 0, (0) | 0.14, (0.38) |
| 4) Did the review authors use a comprehensive literature search strategy? | 0.5, (0) | 0.5, (0) | 0.5, (0) | 0.43, (0.19) |
| 5) Did the review authors perform study selection in duplicate? | 0.5, (0.71) | 0, (0) | 0.33, (0.58) | 0.29, (0.49) |
| 6) Did the review authors perform data extraction in duplicate? | 0, (0) | 0, (0) | 0, (0) | 0, (0) |
| 7) Did the review authors provide a list of excluded studies and justify the exclusions? | 0.5, (0.71) | 0.5, (0.58) | 0.33, (0.58) | 0.29, (0.49) |
| 8) Did the review authors describe the included studies in adequate detail? | 0.25, (0.35) | 0, (0) | 0, (0) | 0, (0) |
| 9) Did the review authors use a satisfactory technique for assessing the risk of bias (ROB) in individual studies that were included in the review? | 0.5, (0) | 0.5, (0) | 0.67, (0.29) | 0.5, (0) |
| 10 ) Did the authors report on the sources of funding for the studies included in the SR? | 0, (0) | 0, (0) | 0.33, (0.29) | 0.07, (0.19) |
| 11) If meta-analysis was performed, did the review authors use appropriate methods for statistical combination of results? | 0, (0) | 0, (0) | 0, (0) | 0, (0) |
| 12) If meta-analysis was performed, did the SR authors assess the potential impact of RoB in individual studies on the results of the meta-analysis/other evidence synthesis? | 0, (0) | 0, (0) | 0, (0) | 0, (0) |
| 13) Did the review authors account for RoB in individual studies when interpreting/ discussing the results of the review? | 1, (0) | 1, (0) | 0.5, (0.71) | 1, (0) |
| 14) Did the review authors provide a satisfactory explanation for, and discussion of, any heterogeneity observed in the results of the review? | 0, (0) | 0, (0) | 0, (0) | 0.5, (0.55) |
| 15) If they performed quantitative synthesis did the review authors carry out an adequate investigation of publication bias (small study bias) and discuss its likely  impact on the results of the review? | 0, (0) | 0, (0) | 0.33, (0.58) | 0.43, (0.53) |
| 16) Did the review authors report any potential sources of conflict of interest, including any funding they received for conducting the review? | 0.5, (0.71) | 0.75, (0.5) | 0.33, (0.58) | 0.57, (0.53) |
| AMSTAR-2 Percent Complete | 35.94. (11.05) | 29.93. (13) | 31.65. (7.59) | 39.7. (11.87) |

| Supplementary Table 3 Continued | | | | |
| --- | --- | --- | --- | --- |
| **AMSTAR-2 Item** | **Diagnosis and treatment of melanoma. European consensus-based interdisciplinary guideline - Update 2016**  **(SRs=3)** | **Screening for Skin Cancer: US Preventive Services Task Force Recommendation Statement**  **(SRs=1)** | **Updated evidence-based clinical practice guidelines for the diagnosis and management of melanoma: definitive excision margins for primary cutaneous melanoma**  **(SRs=3)** | **Guidelines of care for the management of primary cutaneous melanoma**  **(SRs=13)** |
|  | Item Score,  Mean (SD) | Item Score,  Mean (SD) | Item Score,  Mean (SD) | Item Score,  Mean (SD) |
| 1) Did the research questions and inclusion criteria for the review include the components of PICO? | 1, (0) | 1, (0) | 1, (0) | 0.85, (0.38) |
| 2) Did the SR contain an explicit statement that the methods were established prior to the SR and did the report justify any significant deviations from the protocol? | 0.33, (0.58) | 0, (0) | 0.33, (0.58) | 0.04, (0.14) |
| 3) Did the SR authors explain their selection of the study designs for inclusion? | 0, (0) | 0, (0) | 0.33, (0.58) | 0, (0) |
| 4) Did the review authors use a comprehensive literature search strategy? | 0.5, (0) | 0.5, (0) | 0.5, (0) | 0.5, (0) |
| 5) Did the review authors perform study selection in duplicate? | 0, (0) | 0, (0) | 0.33, (0.58) | 0.77, (0.44) |
| 6) Did the review authors perform data extraction in duplicate? | 0, (0) | 1, (0) | 1, (0) | 0.38, (0.51) |
| 7) Did the review authors provide a list of excluded studies and justify the exclusions? | 0.67, (0.58) | 0, (0) | 0, (0) | 0.15, (0.38) |
| 8) Did the review authors describe the included studies in adequate detail? | 0, (0) | 0.5, (0) | 0.67, (0.29) | 0.58, (0.19) |
| 9) Did the review authors use a satisfactory technique for assessing the risk of bias (ROB) in individual studies that were included in the review? | 0.5, (0) | 0, (0) | 0.33, (0.29) | 0.31, (0.33) |
| 10 ) Did the authors report on the sources of funding for the studies included in the SR? | 0.17, (0.29) | 0, (0) | 0, (0) | 0, (0) |
| 11) If meta-analysis was performed, did the review authors use appropriate methods for statistical combination of results? | 0, (0) | 0, (0) | 0.67, (0.58) | 0.86, (0.38) |
| 12) If meta-analysis was performed, did the SR authors assess the potential impact of RoB in individual studies on the results of the meta-analysis/other evidence synthesis? | 0, (0) | 0, (0) | 0.67, (0.58) | 0.71, (0.49) |
| 13) Did the review authors account for RoB in individual studies when interpreting/ discussing the results of the review? | 1, (0) | 0, (0) | 0.67, (0.58) | 0.54, (0.52) |
| 14) Did the review authors provide a satisfactory explanation for, and discussion of, any heterogeneity observed in the results of the review? | 0.5, (0.71) | 1, (0) | 0.67, (0.58) | 0.46, (0.52) |
| 15) If they performed quantitative synthesis did the review authors carry out an adequate investigation of publication bias (small study bias) and discuss its likely  impact on the results of the review? | 0.33, (0.58) | 0, (0) | 0.33, (0.58) | 0.43, (0.53) |
| 16) Did the review authors report any potential sources of conflict of interest, including any funding they received for conducting the review? | 0.67, (0.58) | 0, (0) | 0.67, (0.58) | 0.92, (0.28) |
| AMSTAR-2 Percent Complete | 41.59. (25.19) | 25. (0) | 51.04. (14.09) | 44.32. (14.99) |

| Supplementary Table 3 Continued | | | | |
| --- | --- | --- | --- | --- |
| **AMSTAR-2 Item** | **Cutaneous Melanoma, Version 2.2019, NCCN Clinical Practice Guidelines in Oncology**  **(SRs=3)** | **Update on Current Treatment Recommendations for Primary Cutaneous Melanoma**  **(SRs=4)** | **Primary excision margins, sentinel lymph node biopsy, and completion lymph node dissection in cutaneous melanoma: a clinical practice guideline**  **(SRs=4)** | **Evidence-Based Clinical Practice Guidelines for the Management of Patients with Lentigo Maligna**  **(SRs=4)** |
|  | Item Score,  Mean (SD) | Item Score,  Mean (SD) | Item Score,  Mean (SD) | Item Score,  Mean (SD) |
| 1) Did the research questions and inclusion criteria for the review include the components of PICO? | 0.33, (0.58) | 1, (0) | 1, (0) | 1, (0) |
| 2) Did the SR contain an explicit statement that the methods were established prior to the SR and did the report justify any significant deviations from the protocol? | 0, (0) | 0.25, (0.5) | 0.5, (0.58) | 0.38, (0.48) |
| 3) Did the SR authors explain their selection of the study designs for inclusion? | 0, (0) | 0, (0) | 0.25, (0.5) | 0, (0) |
| 4) Did the review authors use a comprehensive literature search strategy? | 0.17, (0.29) | 0.5, (0) | 0.63, (0.25) | 0.63, (0.25) |
| 5) Did the review authors perform study selection in duplicate? | 0.33, (0.58) | 0.75, (0.5) | 0.5, (0.58) | 1, (0) |
| 6) Did the review authors perform data extraction in duplicate? | 0.33, (0.58) | 0.75, (0.5) | 1, (0) | 0.5, (0.58) |
| 7) Did the review authors provide a list of excluded studies and justify the exclusions? | 0, (0) | 0.25, (0.5) | 0, (0) | 0.25, (0.5) |
| 8) Did the review authors describe the included studies in adequate detail? | 0.5, (0) | 0.75, (0.29) | 0.38, (0.25) | 0.75, (0.29) |
| 9) Did the review authors use a satisfactory technique for assessing the risk of bias (ROB) in individual studies that were included in the review? | 0.17, (0.29) | 0.5, (0.41) | 0.38, (0.25) | 0.5, (0.58) |
| 10 ) Did the authors report on the sources of funding for the studies included in the SR? | 0, (0) | 0.25, (0.5) | 0, (0) | 0.25, (0.5) |
| 11) If meta-analysis was performed, did the review authors use appropriate methods for statistical combination of results? | 0.67, (0.58) | 0.75, (0.5) | 1, (0) | 0, (0) |
| 12) If meta-analysis was performed, did the SR authors assess the potential impact of RoB in individual studies on the results of the meta-analysis/other evidence synthesis? | 0.33, (0.58) | 1, (0) | 1, (0) | 0, (0) |
| 13) Did the review authors account for RoB in individual studies when interpreting/ discussing the results of the review? | 0, (0) | 1, (0) | 0.75, (0.5) | 0.5, (0.58) |
| 14) Did the review authors provide a satisfactory explanation for, and discussion of, any heterogeneity observed in the results of the review? | 0.67, (0.58) | 0.5, (0.58) | 1, (0) | 0.25, (0.5) |
| 15) If they performed quantitative synthesis did the review authors carry out an adequate investigation of publication bias (small study bias) and discuss its likely  impact on the results of the review? | 0.33, (0.58) | 0.67, (0.58) | 1, (0) | 0, (0) |
| 16) Did the review authors report any potential sources of conflict of interest, including any funding they received for conducting the review? | 0.67, (0.58) | 1, (0) | 1, (0) | 1, (0) |
| AMSTAR-2 Percent Complete | 28.13. (27.24) | 62.17. (20.29) | 61.78. (19.55) | 53.85. (29.46) |

| Supplementary Table 3 Continued | | | | | |
| --- | --- | --- | --- | --- | --- |
| **AMSTAR-2 Item** | **SEOM clinical guideline for the management of cutaneous melanoma (2020)**  **(SRs=3)** | **NCCN Guidelines® Insights: Melanoma: Cutaneous, Version 2.2021**  **(SRs=1)** | **Overall**  **(SRs=36)** | **Cochrane**  **(SRs=4)** | **Non-Cochrane**  **(SRs=31)** |
|  | Item Score,  Mean (SD) | Item Score,  Mean (SD) | Item Score,  Mean (SD) | Item Score,  Mean (SD) | Item Score,  Mean (SD) |
| 1) Did the research questions and inclusion criteria for the review include the components of PICO? | 1, (0) | 1, (0) | 0.81, (0.4) | 1, (0) | 0.78, (0.42) |
| 2) Did the SR contain an explicit statement that the methods were established prior to the SR and did the report justify any significant deviations from the protocol? | 0.33, (0.58) | 0, (0) | 0.15, (0.35) | 1, (0) | 0.05, (0.2) |
| 3) Did the SR authors explain their selection of the study designs for inclusion? | 0.33, (0.58) | 0, (0) | 0.11, (0.32) | 0.25, (0.5) | 0.09, (0.3) |
| 4) Did the review authors use a comprehensive literature search strategy? | 0.5, (0) | 0.5, (0) | 0.49, (0.19) | 0.75, (0.29) | 0.45, (0.15) |
| 5) Did the review authors perform study selection in duplicate? | 0.67, (0.58) | 1, (0) | 0.53, (0.51) | 1, (0) | 0.47, (0.51) |
| 6) Did the review authors perform data extraction in duplicate? | 0.33, (0.58) | 1, (0) | 0.53, (0.51) | 1, (0) | 0.47, (0.51) |
| 7) Did the review authors provide a list of excluded studies and justify the exclusions? | 0.33, (0.58) | 0, (0) | 0.15, (0.35) | 0.75, (0.5) | 0.08, (0.26) |
| 8) Did the review authors describe the included studies in adequate detail? | 0.67, (0.29) | 0.5, (0) | 0.58, (0.22) | 0.88, (0.25) | 0.55, (0.2) |
| 9) Did the review authors use a satisfactory technique for assessing the risk of bias (ROB) in individual studies that were included in the review? | 0.33, (0.58) | 0.5, (0) | 0.28, (0.35) | 0.88, (0.25) | 0.2, (0.28) |
| 10 ) Did the authors report on the sources of funding for the studies included in the SR? | 0, (0) | 0, (0) | 0.06, (0.23) | 0.5, (0.58) | 0, (0) |
| 11) If meta-analysis was performed, did the review authors use appropriate methods for statistical combination of results? | 1, (0) | 1, (0) | 0.84, (0.37) | 1, (0) | 0.82, (0.39) |
| 12) If meta-analysis was performed, did the SR authors assess the potential impact of RoB in individual studies on the results of the meta-analysis/other evidence synthesis? | 1, (0) | 1, (0) | 0.5, (0.51) | 1, (0) | 0.45, (0.51) |
| 13) Did the review authors account for RoB in individual studies when interpreting/ discussing the results of the review? | 1, (0) | 1, (0) | 0.42, (0.5) | 1, (0) | 0.34, (0.48) |
| 14) Did the review authors provide a satisfactory explanation for, and discussion of, any heterogeneity observed in the results of the review? | 0.33, (0.58) | 1, (0) | 0.64, (0.49) | 1, (0) | 0.59, (0.5) |
| 15) If they performed quantitative synthesis did the review authors carry out an adequate investigation of publication bias (small study bias) and discuss its likely  impact on the results of the review? | 0.67, (0.58) | 1, (0) | 0.46, (0.51) | 1, (0) | 0.41, (0.5) |
| 16) Did the review authors report any potential sources of conflict of interest, including any funding they received for conducting the review? | 1, (0) | 1, (0) | 0.78, (0.42) | 1, (0) | 0.75, (0.44) |
| AMSTAR-2 Percent Complete | 59.38. (21.88) | 65.63. (0) | 44.55. (21.05) | 86.8. (4.94) | 40.04. (15.07) |
